# New insights about endometriosis-associated ovarian cancer: pathogenesis, risk factors, prediction and diagnosis and treatment

**DOI:** 10.3389/fonc.2024.1329133

**Published:** 2024-02-07

**Authors:** Biqing Chen, Liping Zhao, Rulin Yang, Tianmin Xu

**Affiliations:** Department of Obstetrics and Gynecology, The Second Hospital of Jilin University, Changchun, China

**Keywords:** EAOC, ovarian cancer, pathogenesis, diagnosis, treatment

## Abstract

Previous studies have shown that the risk of malignant transformation of endometriosis in premenopausal women is approximately 1%, significantly impacting the overall well-being and quality of life of affected women. Presently, the diagnostic gold standard for endometriosis-associated ovarian cancer (EAOC) continues to be invasive laparoscopy followed by histological examination. However, the application of this technique is limited due to its high cost, highlighting the importance of identifying a non-invasive diagnostic approach. Therefore, there is a critical need to explore non-invasive diagnostic methods to improve diagnostic precision and optimize clinical outcomes for patients. This review presents a comprehensive survey of the current progress in comprehending the pathogenesis of malignant transformation in endometriosis. Furthermore, it examines the most recent research discoveries concerning the diagnosis of EAOC and emphasizes potential targets for therapeutic intervention. The ultimate objective is to improve prevention, early detection, precise diagnosis, and treatment approaches, thereby optimizing the clinical outcomes for patients.

## 1 Introduction

Endometriosis is a persistent, non-malignant inflammatory ailment that is subject to estrogenic influence and frequently manifests in conjunction with chronic pelvic pain, dysmenorrhea, and infertility. It is estimated to impact around 5-15% of women in their reproductive years (1). Although endometriosis is typically categorized as a benign condition, it exhibits biological characteristics akin to malignant tumors, including rapid growth, extensive proliferation, angiogenesis. A previous cohort study has shown that the prevalence of ovarian cancer in women with endometriosis is 1.37 times higher compared to the general population (2). Furthermore, previous research has indicated that the occurrence of malignant transformation in premenopausal women with endometriosis is approximately 1%, while the likelihood of malignant transformation in postmenopausal women ranges from 1-2.5% (3). The connection between ovarian endometriosis and EAOC is believed to be established through the development of endometrial cysts within the ovary (4). Atypical endometriosis (AE) serves as an intermediary stage in which benign lesions evolve into malignant lesions. Women who have a prolonged history of endometriosis are at a heightened risk of developing EAOC, especially if the duration of the disease surpasses 10 years after the initial diagnosis of endometriosis or if there is a frequent occurrence of ovarian endometriosis (5). It is widely acknowledged that the occurrence of EAOC is atypical in instances of ovarian endometriosis, particularly in the clear cell and endometrial subtypes (6, 7).

In 1925, Sampson first outlined the diagnostic criteria for EAOC (8). The etiology of EAOC is commonly ascribed to a variety of complex pathogenic factors, such as endocrine dysregulation, oxidative stress, immune dysregulation, and intricate changes in immune surveillance, ultimately resulting in chronic inflammation (9). The primary objective of this article is to present a thorough examination of the recent progress made in comprehending the pathogenesis of endometriosis malignant transformation. Furthermore, it will explore the most recent research pertaining to the identification of early-stage EAOC, with the ultimate aim of improving prevention, early detection, precise diagnosis, and treatment approaches. We conducted a comprehensive search of the pubmed database to identify research articles pertaining to endometriosis-associated ovarian cancer (EAOC) within the last five years. The search terms employed were “endometriosis malignant transformation” and “endometriosis-associated ovarian cancer.” Only articles presenting complete experimental data and conclusive findings were considered for inclusion, while those with ambiguous or inconclusive research outcomes were excluded.

## 2 Pathogenesis of EAOC

### 2.1 Abnormal expression of related genes

Multiple studies suggest that ARID1A may act as a tumor suppressor (10). In their study, Guan et al. made the significant finding that ARID1A operates as a tumor suppressor and engages in an interaction with the P53 protein, thereby impeding cell proliferation through the p53-dependent transcriptional regulation of CDKN1A and SMAD3. The mutations in P53 or ARID1A impede the transcription of tumor suppressors, thereby causing uncontrolled cell proliferation and ultimately resulting in EAOC (11). Recent genomic research and targeted analysis have unveiled frequent mutations in the ARID1A and PIK3CA genes in ovarian clear cell carcinoma, with moderate mutations observed in PPP2R1A and KRAS (12). Similarly, endometrial carcinoma has been discovered to manifest mutations in PTEN, CTNNB1, and KRAS (13). These findings, when amalgamated with gene expression profiling, suggest the activation of the KRAS and PI3K survival pathways and the deactivation of tumor suppressor genes PTEN and ARID1A in clear cell and endometrioid ovarian cancers. Moreover, it is noteworthy that the lack of ARID1A expression, as detected by immunohistochemical analysis, could potentially be associated with ARID1A truncating mutations (14).

Furthermore, the lack of p53 has been observed to lead to an exaggerated proliferation of endometrial glands (15). ARID1A mutations has been hypothesized that this mutation plays a pivotal role as an initial molecular event in the progression of EAOC (16). Prior research has suggested that the presence of ARID1A somatic mutation and subsequent absence of BAF250a protein do not demonstrate a correlation between endometriosis and the ovarian response to chemotherapy (6). The presence of BAF250a is highly correlated with the early stages of carcinogenesis in endometriosis. The lack of ARID1A has been associated with a higher presence of CD8+ tumor-infiltrating lymphocytes (TILs) and intratumoral CD8+ immune cells in EAOC, suggesting the potential effectiveness of targeted immunotherapy in this specific context (17). Furthermore, it has been suggested that the inclusion of supplementary driver events may be imperative for the transformation of ovarian endometriosis with ARID1A loss-of-function mutations (18).

Multiple studies have provided evidence of an increase in the copy number of the CCNE1 gene and an up-regulation of CCNE1 in ovarian clear cell carcinoma. Cyclin E1, in conjunction with the regulatory subunit cyclin-dependent kinase 2 (Cdk2), plays a crucial role in facilitating the transition of the cell cycle from the G1 phase to the S phase. While normal cells tightly regulate cyclin E1 activity, cancer cells exploit its upregulation to enhance the replication of tumor cells. This phenomenon is particularly observed in clear cell carcinomas within EAOC (19).

The frequent activation of the PI3K/AKT pathway in endometrioid and ovarian clear cell carcinomas is a result of mutations in PIK3CA, AKT, and PTEN, leading to their inactivation (20). The presence of PIK3CA mutation, which activates the PI3K/AKT pathway, and the loss of PTEN expression have been extensively documented in around 33 to 40% of ovarian clear cell carcinomas and 40% of endometrioid carcinomas (21, 22). Guan et al. demonstrated that alterations in the PI3K/PTEN/AKT pathway are necessary prerequisites for promoting tumor progression (11). In a separate publication, Gounaris et al. identified the inactivation of the PIK3CA-mTOR and RAS-RAF-MAPK pathways in the eutopic endometrium of endometriosis as a significant contributing factor to the malignant transformation associated with endometriosis (23). Previous studies have provided evidence indicating the advantageous role of Met gene amplification in promoting the malignant transformation of endometriosis. The Met/PI3K/AKT pathway signal plays a significant role in the progression of malignant transformation. Therefore, targeted inhibition of the Met pathway emerges as a potentially promising therapeutic approach for EAOC (24).

The early progression of endometriosis involves the inactivation of the tumor suppressor gene protein phosphatase and tension homologue (PTEN) at locus 10q23.3, as identified in previous research (25). This inactivation is a result of the loss of heterozygosity at locus 10q23.3 and mutation of PTEN, subsequently leading to the activation of the phosphatidylinositol 3-kinase (PI3K) -protein kinase B (AKT) -mammalian target of rapamycin (mTOR) signaling pathway (26). In the context of endometriosis, atypical endometriosis, and EAOC, the frequent occurrence of loss of heterozygosity resulting in PTEN inactivation suggests a potential continuum between endometriosis and ovarian cancer. Moreover, the presence of somatic mutations in the PTEN gene is highly prevalent in ovarian endometrioid adenocarcinoma, but uncommon in other pathological subtypes (27). Consequently, PTEN has the potential to function as a distinctive molecular alteration in EAOC.

The upregulation of Fibroblast growth factor receptor 2 (FGFR2) expression in ovarian endometriosis demonstrates aberrant elevation during the progression towards malignancy (28). This anomalous expression can be attributed to the occurrence of alternative splicing events within the FGFR2 gene, specifically involving the epithelial FGFR2IIIb subtype (encoded by exon 8) and the mesenchymal FGFR2IIIc subtype (utilizing exon 9). Furthermore, Steele et al. have demonstrated that ligands for FGFR2IIIb have a notable impact on various phenotypes that play a critical role in the growth of epithelial ovarian cancer cells (29). Furthermore, it has been postulated that autocrine FGF7 and paracrine FGF10 signaling cascades could be involved in the augmented epithelial differentiation observed during the course of malignant transformation. Specifically, the upregulation of FGFR2 expression holds the capacity to trigger excessive FGFR2 signal transduction, potentially playing a role in the pathogenesis of endometriosis. Moreover, targeting FGFR2 may present a promising therapeutic strategy for impeding the malignant advancement of endometriosis-associated cancer (refer to **Table 1**).

**Table 1:** Pathogenesis of ovarian cancer associated with endometriosis: abnormal expression of related genes.

Gene	Mechanism	Results	References
ARID1A	Mutation deactivation	Inhibition of the transcription of tumor suppressor factors allows cell proliferation	(10, 11)
PIK3CA	Mutation	The activation of PI3K survival pathway	(12)
KRAS	Mutation	The activation of KRAS survival pathway	(12, 13)
PTEN	Inactivation of tumor suppressor gene mutations	Proliferation of cells	(13, 25–27)
PPP2R1A	Mutation	Proliferation of cells	(12)
CTNNB1	Mutation	Proliferation of cells	(13)
P53	Deletion	Proliferation of cells	(15)
BAF250a	Deletion	It is involved in the early carcinogenesis process	(6)
ARID1A	Deletion	Mismatch repair deficiency and increased CD8+ tumor-infiltrating lymphocytes	(17)
CCNE1	A rise in gene copy number increase and CCNE1	It is involved in cell cycle regulation	(19)
AKT	PI3K/AKT pathway activation	Proliferation of cells	(20–22)
Met	Gene amplification	Met/PI3K/AKT pathway activation	(24)
FGFR2	High expression	Autocrine FGF7 and paracrine FGF10 signal ring	(28, 29)

### 2.2 Genetic regulation of miRNA

MicroRNAs (miRNAs) are essential regulators of gene expression. They play a crucial role in functioning as either oncogenes or tumor suppressor genes. Conserved non-coding RNAs, which serve as regulators of target mRNA expression or degradation, have been recognized as potentially influential factors in the malignant transformation of endometriosis (30). As a result, these microRNAs (miRNAs) show potential as biomarkers for both endometriosis and EAOC. The simultaneous evaluation of multiple biomarkers can greatly improve the prognostic predictive value, indicating that a panel of miRNAs may offer a more dependable indicator of disease.

The miR-200 family, particularly miR-200-a and miR-200-b, have garnered significant attention in the field of endometriosis research. Notably, Ohlsson et al. conducted a study that demonstrated a noteworthy decrease in the expression of the miR-200 family, which subsequently led to the occurrence of epithelial-mesenchymal transition, a distinctive hallmark of endometriosis (31). The reduction in ARID1A expression may play a crucial role in the advancement of EAOC in patients who display heightened levels of miR-221 and miR-222 (20). Additional research is necessary to investigate the potential of miR-222 and miR-221 as biomarkers for EAOC. Furthermore, it was observed that miR-143 exhibited upregulation in the serum of patients with EAOC, thereby correlating with heightened cell invasion and migration. This augmented expression of miR-143 consequently results in the suppression of transcription of its target gene FNDC3B, a known facilitator of cell invasion and migration (32).

The association between the cycle of endometriosis and biomarker miR-20a has been extensively studied. Research has provided evidence for the significant role of miR-20a in the pathogenesis of endometriosis, as it directly targets TGF-β and Il-8 (33). A decrease in miR-20a expression results in elevated levels of these cytokines, which may contribute to the promotion of inflammation and tissue repair. By targeting miR-20a to inhibit TGF-β and Il-8, a better understanding of the development of endometriosis lesions could potentially be achieved. It is worth mentioning that miR-20a exhibits up-regulation in ovarian tissues of individuals diagnosed with ovarian endometriosis, thereby playing a role in neovascularization (34). Furthermore, the down-regulation of several mirnas, such as miR-3613-5p, miR-6755-3p (35), let7b, miR-125a (36), and others, has been observed in EAOC tissues. The investigation has provided evidence that miR-191 plays a direct regulatory role in the expression of TIMP3, thereby influencing cellular proliferation and invasion. TIMP3, a pro-apoptotic protein, exhibits an inverse correlation with cell growth and invasion (37, 38).

### 2.3 Oxidative stress

The recurrent hemorrhaging and accumulation of heme and free iron within endometriotic lesions are hypothesized to exert a substantial influence on the initiation of ovarian cancer, primarily through the production of reactive oxygen species (ROS) (39). Yamaguchi et al. have reported the high concentration of iron in endothelial cell fluid, leading to the induction of oxidative stress (5, 40). Recent studies have underscored the importance of the interaction between oxidative stress and non-coding miRNAs in the advancement of EAOC (41). *In vitro* investigations have revealed that endometriotic cyst contents manifest an elevated production of ROS and a heightened inclination to elicit gene mutations in comparison to other cyst contents (5). Correspondingly, Sanchez et al. have observed the existence of markers denoting oxidative damage, such as strand breaks, DNA adducts, and lipid peroxidation products, in ovarian cancer tissues (42, 43). The gene expression profile obtained from microarray analysis further substantiates the correlation between oxidative stress and ovarian cancer, particularly in the context of clear cell carcinoma progression (44).

A considerable percentage of the genes displaying elevated expression levels in ovarian clear cell carcinoma are linked to redox processes, including oxidative and detoxification enzymes (45). HNF-1β, acting as a transcription factor, exerts control over target genes responsible for encoding proteins involved in vital cellular processes such as proliferation, differentiation, glucose metabolism, dysplasia, and glycogen synthesis (46). In the domain of ovarian cancer, Liu et al. conducted a study employing the cut HNF-1 beta shRNA strategy, which exhibited heightened susceptibility of ovarian cancer cells to cisplatin and paclitaxel-induced cytotoxicity, both *in vitro* and *in vivo* (47).The accumulation of excessive free radicals can lead to cellular harm and eventual cell demise, whereas the persistent exposure to sublethal ROS, combined with an improved antioxidant status, has the potential to amplify the tumorigenicity of endometriotic cells (48). In a specific study, the utilization of enzyme-linked immunosorbent assay was employed to examine cyst fluid samples collected from a total of 44 patients diagnosed with ovarian endometriosis (OE) and 14 patients diagnosed with EAOC. The expression level of HO-1 is notably reduced in the EAOC group in comparison to the benign OE group, as indicated by the diminished presence of 8-hydroxy deoxyguanosine (8-OHdG) in the fluid. In contrast, the EAOC group demonstrates heightened levels of antioxidants and heme iron in the fluid in comparison to the OE group. It is worth mentioning that HO-1 exhibits the most significant diagnostic efficacy in discerning between benign and malignant cystic fluid, indicating a robust correlation between REDOX imbalance and the malignant progression of endometriosis (49).

The isoforms of GSTM1 are essential in the process of detoxifying harmful substances. Individuals without GSTM1 may have a greater risk of malignant transformation in endometriotic lesions due to insufficient elimination of oxidative stress products (50). Hydroxy-2’-deoxyguanosine (8-OHdG) has emerged as a potential biomarker with promise for evaluating oxidative DNA damage in various disease states. Within the specific context of endometriotic tissues, the up-regulation of 8-OHdG expression has been observed in EAOC when compared to OE. Additionally, CD44, a cell surface receptor responsible for binding to hyaluronic acid, has been demonstrated a potential protective function against DNA damage induced by ROS. The increased production of reduced glutathione synthesis, is accountable for the activation of CD44, specifically the variant isoform (CD44v). In contrast to OE and EAOC endometriotic tissues, a decrease in CD44v expression is evident in EAOC tumor tissues. This decrease, coupled with alterations in CD44v and 8-OHdG, could potentially be associated with the malignant progression of endometriosis (51). The findings of previous studies have provided evidence that electron microscopic replicas of malignant endometriosis cells display mitochondrial swelling and vacuolar alterations, which suggest the possibility of endometriosis lesions growing in a hypoxic microenvironment. These observations imply that the adverse impact of hypoxia on mitochondria could potentially contribute to an increased probability of malignant transformation (52).

### 2.4 Abnormal gene methylation

Epigenetic modifications, including DNA methylation, histone modifications, and noncoding microRNAs, have emerged as noteworthy factors in the development of EAOC (53), exerting regulatory influence on gene expression independent of alterations in the DNA sequence. Among these modifications, DNA methylation has been extensively studied, with the DNA methyltransferase (DNMT) family playing a pivotal role. Aberrant gene expression and subsequent tumorigenesis can be facilitated by low levels of methylation in cancer gene promoter regions (54). Various studies have demonstrated the involvement of specific genes, such as E-cadherin (CDH1), p16, PTEN, and PTEN hypermethylation in the promoter region, in promoting the malignant transformation of endometriosis (55, 56).

On the other hand, the anomalous hypomethylation of the promoter regions of long interspersed element-1 (LINE-1) (57) and syncytin-1 (58) has been linked to the malignant conversion of endometriosis. The elevated methylation of the hMLH1 promoter region results in the lack of hMLH1 protein expression, a vital constituent of the DNA mismatch repair (MMR) system. This deviation is highly correlated with the malignant advancement of endometriosis (59). The combination of Methylated CpG island amplification and representative difference analysis (DDA) has enabled the discovery of nine candidate genes, namely RASSF2, SPOCK2, RUNX3, GSTZ1, CYP2A, GBGT1, NDUFS1, ADAM22, and TRIM36, that exhibit distinctive methylation patterns associated with the malignant transformation of ovarian endometriosis (60). The transcription factor Runx-related transcription factor 3 (RUNX3), a member of the Runx protein family, plays a crucial role in regulating the self-renewal, proliferation, and differentiation mechanisms (61). Nevertheless, the current literature presents contradictory results regarding the specific function of RUNX3 in ovarian cancer. For instance, Nevadunsky et al. reported a significant upregulation of RUNX3 and its involvement in promoting the proliferation of epithelial ovarian cancer cells (62). Moreover, Barghout et al. have provided evidence of a significant association between the upregulation of RUNX3 and resistance to RBMO chemotherapy in ovarian cancer cases (63). Conversely, alternative investigations have suggested that the hypomethylation and expression of the RUNX3 gene in epithelial ovarian cancer tissue and cell lines are linked to an unfavorable prognosis (64). Furthermore, these studies have corroborated a positive correlation between elevated RUNX3 methylation and the expression of ER alpha (65). One study proposes that the hypomethylation of the estrogen receptor (ESR) β promoter may potentially contribute to the development of progesterone resistance in individuals with endometriosis (66). Another study reveals a complete absence of ESR and PGR in the EAOC organization (67). However, the present study did not detect any significant alterations in the methylation of ESR and PGR genes when subjected to analysis using MCA - RDA (68).

The tumor suppressor gene RASSF2, which has been recently identified, exerts a notable influence on the Ras signaling pathways. Otsuka et al. have reported that the dysregulated activation of Ras genes leading to the upregulation of RASSF2 may play a pivotal role in the malignant transformation of endometriosis (69, 70). Moreover, Fauvet et al. have emphasized that the activation of the K-ras gene, an oncogene implicated in the Ras signaling pathway, may potentially manifest at a subsequent phase during the progression of malignant transformation in ovarian endometriosis (71). The findings of the study reveal a noteworthy discrepancy in the prevalence of RASSF2 promoter hypermethylation between tumor tissues and ectopic endometrial tissues, with a considerably higher incidence observed in the former. The results of this study indicate that the hypermethylation of the RASSF2 promoter, leading to epigenetic inactivation, may play a crucial role in the early stages of ovarian endometriosis progressing towards malignancy (60) (refer to **Table 2**).

**Table 2:** Epigenetic modifications of gene methylation that occur during malignant transformation of endometriosis.

Gene	Mechanism	Results	References
E-cadherin gene	The promoter region was hypermethylated	Promote the malignant transformation of endometriosis	(55)
p16	The promoter region was hypermethylated	Promote the malignant transformation of endometriosis	(56)
PTEN	The promoter region was hypermethylated	Promote the malignant transformation of endometriosis	(56)
LINE-1	Low methylation in the promoter region of the	Malignant transformation of endometriosis	(57)
syncytin-1	Low methylation in the promoter region of the	Malignant transformation of endometriosis	(58)
hMLH1	The promoter region was hypermethylated	Loss of hMLH1 protein expression, malignant transformation of endometriosis	(59)
RUNX3	Hypermethylation	Poor prognosis	(64)
Estrogen receptor (ESR) beta	Low methylation	Absence of ESR	(66, 68)
RASSF2	The promoter region was hypermethylated	Inactivation of genes	(60)

### 2.5 Imbalance in hormonal regulation

The absence of progesterone protection in the context of persistent estrogen stimulation presents a potential hazard for the emergence of malignancy in endometriosis (72, 73). The probability of malignant transformation was found to be elevated, as evidenced by a previous investigation conducted by Lavery and Gillmer, wherein the administration of non-antagonistic estrogen as a therapeutic intervention resulted in the malignant transformation of residual ectopic endometrial lesions (74). Moreover, endometriosis fosters a microenvironment that facilitates the excessive accumulation of estrogen via diverse mechanisms (75). Although aromatase is usually not present in endometrial tissue, research has revealed heightened levels of aromatase enzyme activity in ectopic endometrial tissue. This activity facilitates the conversion of androstenedione and testosterone from the ovaries and adrenal glands into estrone and estradiol (E2) (25). Additionally, it is important to acknowledge that ectopic endometrial tissue lacks the enzyme 17β-hydroxysteroid dehydrogenase (17β-HSD), which is typically present in eutopic endometrial tissue. This enzyme plays a pivotal role in the conversion of E2 to estrone, a less potent variant of estrogen. Conversely, 17β-HSD is responsible for the conversion of estrone to the more potent E2, and this enzyme is present in endometriotic tissues. Consequently, the presence of 17β-HSD in endometriotic tissues leads to an augmented production and diminished inactivation of locally hyperestrogenic E2, thereby intensifying its cumulative impact (76). It is noteworthy to emphasize that an excess of E2 can stimulate cell proliferation by facilitating the production of cytokines, particularly IL-8 and RANTES (77). Moreover, the activation of E2 triggers the production of PGE2, thereby promoting the proliferation of tumors. Furthermore, it potentially augments the function of aromatase, thus establishing a reinforcing cycle that sustains the continuous accumulation of estrogen in endometriosis (25). An abnormal accumulation of estrogen in the local area contributes to the progression of normal ectopic endometrium towards dysplasia or potentially malignant transformation (2).

In the context of EAOC, the endometrioid subtype is predominantly distinguished by the presence of estrogen receptor (ER) and progesterone receptor (PR) expression, while the clear cell subtype generally lacks ER or PR expression. The occurrence of oxidative stress and inflammation due to recurrent bleeding in endometriosis contributes to DNA methylation, which is linked to reduced ER expression (78–80). Previous studies have provided evidence suggesting that the classical ERα signaling pathway experiences significant inactivity during the transition from endometriosis to EAOC, as demonstrated by the downregulation of genes. In contrast, the gene expression of estrogen-associated ovarian cancer (EAOC) in patients with endometriosis demonstrates features of estrogen resistance, as indicated by notably reduced levels of estrogen receptor alpha (ERα) and progesterone receptor (PR), and elevated levels of estrogen receptor beta (ERβ) compared to individuals with normal endometrium. ERβis widely acknowledged for its antiproliferative properties and its antagonistic impact on ERα-mediated proliferation. The impact of ERαto ERβsignaling on the progression of EAOC from endometriosis is contingent upon the specific tissue context. Furthermore, the de-repression of ERαtarget genes, including FGF18, potentially plays a role in the transformation of endometriosis into EAOC (81).

There is a proposition that progesterone exhibits anti-inflammatory attributes within the endometrium. Prior research employing mouse models has provided evidence that inhibiting ERαorβisoforms, coupled with a concurrent decrease in inflammation, effectively hinders the progression of endometriosis (82, 83). Moreover, recent studies have unveiled a noteworthy association between IL-6 and E2 in the advancement of endometriosis (38). Studies have suggested that the estrogen - DNMT1 signaling pathways potentially contribute to the upregulation of RUNX3 methylation, consequently facilitating the malignant transformation of endometriosis (84). Furthermore, there is a suggestion that hormone replacement therapy (HRT) may have the potential to induce malignant transformation in women with a history of endometriosis (85). The risk of adverse effects increases with prolonged usage of hormone replacement therapy (HRT), especially when exceeding a duration of 10 years (86). It is worth noting that available evidence suggests that the use of estrogen alone carries a higher risk of endometriosis malignant transformation compared to the combined administration of estrogen and progestin (85). In contrast, the utilization of hormone replacement therapy (HRT) did not exhibit a heightened propensity for ovarian cancer in postmenopausal women with a medical history of endometriosis or the development of endometriosis (87).

### 2.6 Imbalance of immune regulation and inflammation

The findings from studies conducted on both human subjects and rats have demonstrated that endometriosis sites display a greater abundance of activated inflammatory cells and cytokines in comparison to the corresponding eutopic endometrium (88). The presence of acute and chronic inflammation is a distinctive characteristic of endometriosis, evident at different stages of tumor advancement, including initiation, malignant transformation, invasion, and metastasis, thereby exerting a substantial impact. Moreover, inflammation disrupts the body’s immune surveillance, resulting in the infiltration of immune cells into tumor tissue and engaging in dynamic interactions with cancer cells.

The literature has provided evidence that in individuals with endometriosis, a notable increase in the population of activated macrophages has frequently been observed in the peritoneal fluid (89). Moreover, there has been an observed elevation in the concentration of various essential cytokines and chemokines, such as TNF alpha, beta, IL-1, IL-6, IL-8, regulated upon activation, normal T cell expressed and secreted (RANTES), and monocyte chemotactic protein 1. The chemotactic agent is present in the latter three, resulting in the accumulation of macrophages (90). Additionally, the presence of ferroportin was detected in the epithelium of ovarian endometrioma and clear cell ovarian cancer, while iron-coated M2 macrophages were identified in the stroma of these conditions. The infiltration of epithelial cells into the stroma of ovarian endometrioma suggests the potential participation of iron-coated M2 macrophages in the carcinogenic process of this ailment (91). Research on the quantity and characteristics of macrophages implicated in the malignant progression of endometriosis has consistently demonstrated a reduction in the expression of the antioxidant marker HO-1 in EAOC. This suggests that a diminished presence of M2 macrophages expressing HO-1 may play a significant role in promoting malignancy (92–94).

The substantial involvement of inflammatory mediators and diverse cytokines, including TNF-α, IL-1β, and IL-6, in the initiation, proliferation, and progression of epithelial ovarian cancer, akin to the observations made in endometriosis (95). Szlosarek et al. investigate the role of TNF alpha in the advancement of ovarian cancer, encompassing serous and clear cell subtypes, and observe heightened expression levels of TNF alpha in comparison to normal ovarian tissue. Moreover, previous studies have reported a substantial upregulation of TNF-α mRNA in cultured ovarian cancer cells (96). Further analysis of the same dataset has demonstrated that this increased expression of TNF network genes within the tumor microenvironment leads to augmented signaling pathways associated with inflammation, and NOTCH signaling (97). The observed up-regulation of small inducible cytokine A2 (SICA2) and small inducible cytokine subfamily A member 14 (CCL14) in endometriosis-associated endometrioid ovarian cancer suggests a notable contribution of inflammatory factors in the pathogenesis of both endometriosis and its associated endometrioid ovarian cancer. Prostaglandin E2 (PGE2), a pivotal mediator of the inflammatory response, has also been shown to exert influence on critical mechanisms linked to tumor growth, including cell proliferation, and inhibition of apoptosis (98).

The Nod-like receptor protein structure domain related protein 3 (NLRP3) inflammatory corpuscle is a multifaceted protein implicated in the innate inflammatory immune response. This intricate assembly encompasses the NLRP3 protein, serving as a detector for inflammasome activation, and the apoptosis-associated speck-like protein containing the CARD complex (ASC). The ASC complex recruits pro-caspase via its CARD domain, thereby facilitating subsequent cascades. The precursor form of caspases is substituted by active caspases, leading to the cleavage of proinflammatory cytokines (precursors of IL-1β and IL-18) into their active states. IL-1β and IL-18, in turn, promote the recruitment of further immune cells associated with inflammation. As a result, the activation of this cancer gene takes place. Consequently, the persistent aseptic inflammation of the NLRP3 signaling pathway potentially functions as the primary phase of carcinogenesis (99). AIM2 functions as a cytoplasmic receptor that identifies double-stranded DNA, particularly originating from viral or bacterial origins, via its carboxyl end hin200 structure domain. This recognition event initiates a series of molecular processes, including the activation of inflammatory proteins and the assembly of AIM2 inflammatory corpuscles. The activation of AIM2 inflammasomes, in conjunction with other conventional inflammasomes, ultimately culminates in inflammatory cell death. In a comparative bioinformatics analysis of endometriosis and ovarian cancer, the immunohistochemical staining analysis further substantiated a robust association between elevated AIM2 expression and heightened Ki-67 activity in clinical samples of EAOC. This discovery lends support to the hypothesis that the alteration of AIM2 and the inflammatory corpuscle in EAOC significantly contribute to the regulation of disease progression.

Anomalous humoral immunity and complement activation significantly contribute to the pathogenesis of EAOC, with cell proliferation serving as a primary mechanism (9). Recent research indicates that there are multiple complement pathways present and operating within the tumor microenvironment, directly stimulating the proliferation of tumor cells and indirectly aiding in immunosuppression and neovascularization (100, 101). The study offers evidence that the activation of Kras and Pten tumor-driven pathways leads to the up-regulation of complement in epithelial cells. The aforementioned findings establish a novel association between the initiation of tumors and immune surveillance facilitated by complement. In conjunction with alterations in immune cells and cytokines, patients with endometriosis commonly manifest heightened activation of B cells. Previous research has established that individuals who have been diagnosed with endometriosis possess the ability to produce systemic antibodies and deposit immunoglobulin G (IgG) and complement in tissues as a humoral response to various autoantigens (102). The mechanism of antibody-induced complement mediated apoptosis efficiently eradicates cells through the classical pathway, which is partially triggered by the attachment of immunoglobulin Fc to infectious agents or diverse antigens found on apoptotic cells. Furthermore, the initiation of this alternative pathway can occur via sequential low-level cleavage of C3 and can be stimulated by various microorganisms such as bacteria, viruses, fungi, and tumor cells. The third complement activation pathway, referred to as the MBL pathway, is activated in response to pathogen-associated molecular patterns (103). Presently, ongoing clinical trials are investigating the focused inhibition of complement as a pharmacological intervention, and the results of these studies will contribute to the development of personalized treatment strategies for patients.

Modifications in immune surveillance might serve as an early indication of the development of cancer in benign conditions (104). Complement signaling can induce diverse immunosuppressive mechanisms, such as the regulation of CD4+ and CD8+ T lymphocytes (105). Furthermore, *in vivo* studies have confirmed the synergistic antitumor impact achieved by combining complement component fragment 5a receptor signaling blockade with PD-L1 antibody, highlighting its reliance on CD8+ T cells (106). On the other hand, the existence of infiltrating T lymphocytes (ITLs), including CD8 + T cells, regulatory T cells, regulatory B cells type II natural killer T cells, and Th2 type CD4 + cells, has been linked to tumor remodeling and potentially aiding tumor growth through immunosuppressive mechanisms (107). These cells possess the capability to hinder the host’s anti-tumor response and stimulate angiogenesis within tumors. The impairment of immune cell function and aberrant expression of suppressor T cell response are widely recognized consequences of the interaction between programmed cell death protein-1 (PD-1) and its ligand, programmed cell death ligand-1 (PD-L1), in pathological conditions such as cancer and chronic infection (107). Studies have demonstrated that individuals with endometriosis display elevated levels of PD-1/PD-L1 expression in their circulatory system (108). Moreover, previous research has provided evidence indicating that the upregulation of PD-1/PD-L1 expression occurs in both eutopic and ectopic endometrial tissues among individuals with endometriosis (109). Nevertheless, the precise impact of these immune adaptations on the development and progression of ovarian cancer remains uncertain (108).

**Figure 1:**
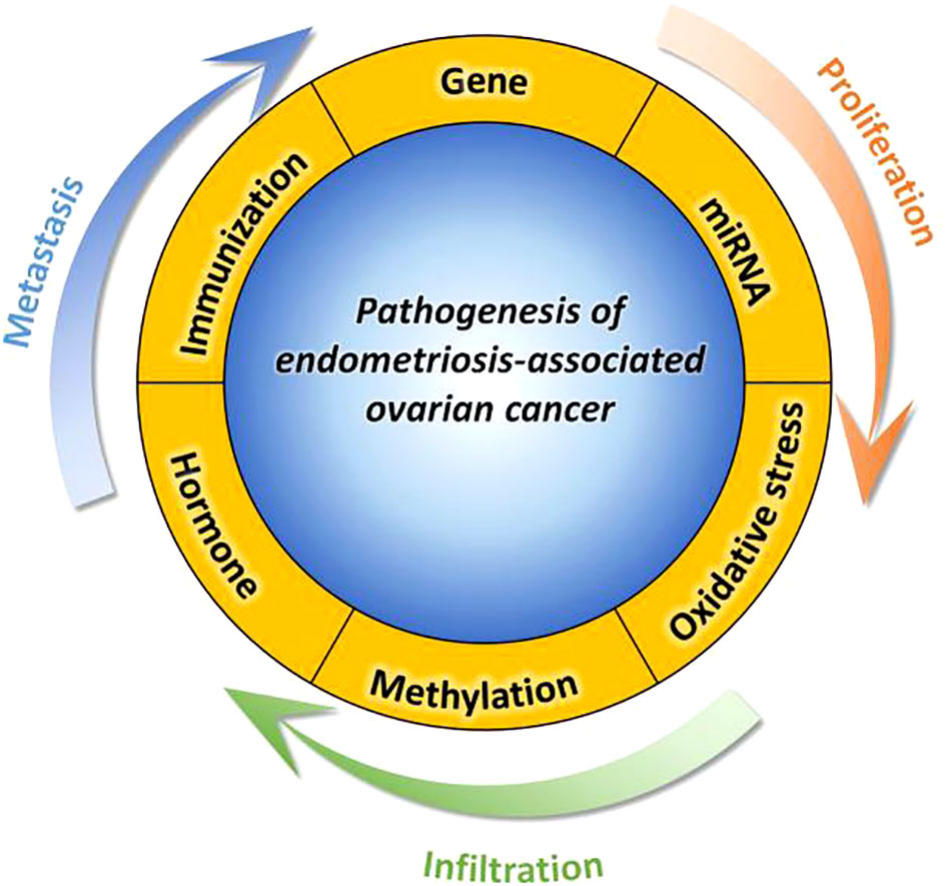
This paper aims to provide a comprehensive understanding of the molecular mechanism involved in the pathogenesis of EAOC by examining six distinct aspects, namely abnormal gene expression, miRNA genetic regulation, oxidative stress, abnormal gene methylation, hormone regulation imbalance, immune regulation imbalance, and inflammation.

Women who have been diagnosed with endometriosis exhibit increased levels of proinflammatory substances, such as tumor necrosis factor-a, interleukin-1b, and interleukin-6. These factors may potentially play a role in the perpetuation of chronic inflammation, thereby promoting the progression and development of EAOC (110, 111). The identification of a heightened frequency of CD8+ cytotoxic T cells has emerged as a promising prognostic determinant in diverse tumor categories, encompassing ovarian cancer (112). These discoveries augment our comprehension of inflammation and immunity as plausible molecular biomarkers for monitoring the advancement of endometriosis towards malignancy, while also offering potential avenues for therapeutic interventions in instances of EAOC. Refer to **Figure 1**.

## 3 Prediction and diagnosis of malignant transformation risk factors

### 3.1 Organizing cytology diagnosis methods

The integration of conventional cytogenetic methods and advanced genetic detection techniques enables the identification of numerical and structural chromosomal abnormalities. However, current literature reports suggest that cytogenetic investigations of individuals with endometriosis often yield inconsistent results. The existing body of research indicates a widely accepted agreement that atypical endometrial hyperplasia encompasses both cellular and structural atypia. However, it is important to note that cellular atypia is more commonly observed in non-cancer patients, whereas structural atypia is more prevalent among patients diagnosed with endometrioid adenocarcinoma (113, 114).

### 3.2 Serological diagnosis methods

Recent retrospective studies have indicated that preoperative CA125 values are not effective in identifying the malignant transformation of endometriosis (115). Prior investigations have suggested that the assessment of CA19-9, CEA, SLX, and LDH serum levels holds promise as valuable indicators for distinguishing between ovarian tumors related to endometriosis and ovarian endometriosis itself in the preoperative evaluation (116). Arakawa et al. conducted a study in which they observed a specific elevation in serum levels of tissue factor pathway inhibitor 2 (TFPI2) in patients diagnosed with ovarian clear cell carcinoma within the subset of individuals with epithelial ovarian cancer (117). The correlation between CTNNB1 and elevated expression of the HIF1A gene suggests disease advancement, particularly during the initial phases (118). Moreover, the stimulation of AMP-activated protein kinase (AMPK) by TSPAN1 has been shown to promote the development of endometriosis and cellular proliferation (119). TSPAN1 has been recognized as a prospective gene candidate for the screening of high-risk endometriosis, thereby facilitating the advancement of therapeutic pharmaceuticals.

### 3.3 Imaging diagnostic methods

The ultrasonography assessment of diverse parameters indicates that the recognition of a “vascular solid component” facilitates a notably precise discrimination between benign and malignant endometrioid cysts (120, 121). The initial phase of EAOC may pose considerable diagnostic difficulties due to the lack of a mural nodule. A study has suggested that the identification of cyst wall nodules measuring over 1.5cm in height and with a maximum diameter surpassing 7.9cm could potentially serve as innovative diagnostic markers for distinguishing between EAOC and benign OE with wall nodules (122). Moreover, a retrospective case-control study revealed that several factors, including advanced age, menopause, weight loss, cyst diameter equal to or exceeding 8.33cm, and the presence of solid areas on ultrasonography, were identified as noteworthy risk factors for EAOC (123). Kobayashi et al. reported an increased vulnerability to malignancy in individuals aged 45 years or older, those undergoing menopause, and those with dimensions of 9cm or larger (124). Moreover, the existence of a solid component within the cyst increases the likelihood of developing ovarian cancer associated with endometrial cysts, in line with the findings presented by Kadan et al. (125). Notably, diagnostic studies employing MRI have demonstrated that EAOC typically manifests as a unilocular mass with a low T2WI signal within the cystic component (126). As a result, MRI shows potential as a valuable tool for distinguishing EAOC from non-EAOC and aiding in preoperative diagnoses.

### 3.4 The development of a diagnostic method

Yang et al. proposed a model that integrates the marker value HE4 and the ADNEX, resulting in increased the discriminatory ability and sensitivity for distinguishing benign from malignant ovarian tumors (127). The application of transvaginal near-infrared (NIR) imaging might provide diagnostic insights into the malignant advancement of endometriosis and could potentially yield further clinical ramifications, and the incorporation of MR relaxation measurements facilitates the identification of conservative therapeutic approaches (128, 129). A pioneering composite optical ultrasound system, employing near infrared guidance and transvaginal ultrasound, is proposed for the purpose of noninvasively quantifying fluid hemoglobin (Hb) levels. The results suggest that metHb is a common form of hemoglobin in benign endometriotic cysts, and the absorption ratio of cyst fluid at 620/580 nm demonstrates significant specificity and positive predictive value. Therefore, it can be utilized as a practical monitoring test for the prompt detection of malignant transformation in endometriosis (130). Reducing the absorption rate at 620/580 nm could potentially facilitate the identification of individuals necessitating prompt monitoring and surgical intervention, thereby underscoring the significance of clinical assessment in cancer patients.

The Endometriotic Neoplasms Algorithm for risk Assessment (e-NARA) index provides a notable level of specificity in distinguishing between EAOC and benign endometriotic cysts (131). The assessment of intracytothelial iron concentration presents a valuable method for predicting and diagnosing EAOC. The application of proton transverse relaxation time (T2) and T2* (R2) and R2* and relaxation rate in magnetic resonance imaging and optical imaging, including magnetic resonance spectrometry, serves as the exclusive imaging technique (132) for the early anticipation of malignant transformation in molten iron and magnetic resonance (NMR) spectrophotometer. Regardless of age, menopausal status, and cyst size, EAOC exhibits lower R2 values and total iron levels compared to benign ovarian endometriosis cysts. The application of R2 values in distinguishing between EAOC and benign ovarian endometriosis cysts has demonstrated promising levels of accuracy, sensitivity, and specificity (133, 134). Numerous studies have retrospectively evaluated the effectiveness of the Copenhagen index (CPH-I), Risk of Ovarian Malignancy Algorithm (ROMA), and R2 prediction index in forecasting the malignant progression of OE. Notably, the CPH index has been identified as the most reliable predictor for postmenopausal patients with malignant tumors, while the R2 prediction index outperforms other indicators in distinguishing malignant tumors for premenopausal individuals (135). Machine learning algorithms have been employed for the purpose of constructing risk models with the objective of forecasting the probability of malignant transformation of endometriosis in patients (136).

## 4 Recent advances in EAOC related treatment

Currently, there is a dearth of established therapeutic interventions for EAOC gene mutations, whereas immunotherapy has exhibited effectiveness in the treatment of EAOC. Extensive clinical trials have been undertaken to investigate the possibility of inhibiting this pathway, encompassing inhibitors that target PI3K, AKT, and mTORC1 (137). In particular, Poly (ADP-ribose) polymerase (PARP) inhibitors have exhibited effectiveness in the treatment of ovarian cancer (138, 139). Anti-VEGF antibodies have been employed in the management of ovarian cancer, including EAOC (140). In a phase 2 clinical trial investigating the efficacy of nivolumab, an anti-PD-1 antibody, for the treatment of platinum-resistant ovarian cancer, the overall response rate was determined to be 15% (141, 142). Furthermore, Lynch syndrome, which is distinguished by germline mutations. Mutations in genes involved in mismatch repair result in a significant prevalence of microsatellite instability, which acts as a biomarker for vulnerability to immune checkpoint inhibitors (143). As a result, individuals diagnosed with clear-cell ovarian cancer associated with Lynch syndrome are more inclined to experience favorable outcomes with the administration of immune checkpoint inhibitors. Preclinical inquiries utilizing cell lines have substantiated the potential of inhibitors that target IL-6/JAK/STAT pathway as a means of therapeutic intervention (144, 145). Moreover, there have been documented reports suggesting that the administration of anti-IL-6 antibody to a mouse model of ovarian clear cell carcinoma leads to enhanced prognosis (146). Moreover, in a mouse model of ovarian clear cell carcinoma lacking the ARID1A gene, the efficacy of combination therapy comprising HDAC6 inhibitors and anti-PD-L1 antibody has been successfully demonstrated (147, 148).Reducing the generation of ROS could potentially aid in the prevention of the malignant progression of endometriosis (149). The findings of a study suggest that exploring the potential of Chk1 inhibitors as a targeted therapy may be a promising treatment approach for patients with clear-cell ovarian cancer, presenting a new opportunity for combination therapy (150).

## 5 Discussion

Long non-coding RNAs (lncRNAs) and post-translational modifications (PTMs), in the pathogenesis of both endometriosis and ovarian cancer has also been suggested (151). Due to the complex nature of this gynecological disorder and its strong association with tumorigenesis, the mechanisms underlying the origin and development of endometriosis are still not fully understood. Vicente Munoz et al. conducted a study in which they identified plasma metabolites in individuals diagnosed with endometriosis (152). The researchers observed heightened levels of valine, foci, choline-containing metabolites, lysine/arginine, and lipoproteins, while the concentrations of creatinine were relatively diminished compared to women without endometriosis (153). The study will contribute to our understanding of the development of malignant transformation. Additionally, it has the potential to provide a new and effective early diagnostic intervention, thereby improving the chances of successful treatment.

## 6 Conclusion

The specific mechanisms and strategies underlying carcinogenesis in EAOC remain unclear. Further research will contribute to a more comprehensive understanding of the progression of EAOC. Improving our understanding of the pathogenesis of EAOC will contribute to the identification of individuals most prone to the malignant transformation of endometriosis lesions. This knowledge will support the creation of efficacious preventive measures for women with endometriosis who are at the greatest risk of developing EAOC, as well as the formulation of innovative therapeutic approaches for those diagnosed with EAOC.

## Author contributions

BC: Writing – original draft. LZ: Conceptualization, Writing – review & editing. RY: Data curation, Writing – review & editing. TX: Writing – review & editing.

## References

[1] Kim HS Kim TH Chung HH Song YS. Risk and prognosis of ovarian cancer in women with endometriosis: a meta-analysis. Br J Cancer (2014) 110(7):1878–90. doi: 10.1038/bjc.2014.29 PMC3974076 24518590

[2] He J Chang W Feng C Cui M Xu T. Endometriosis Malignant transformation: epigenetics as a probable mechanism in ovarian tumorigenesis. Int J Genomics (2018) 2018:1–13. doi: 10.1155/2018/1465348 PMC5892233 29780815

[3] Giannella L Marconi C Di Giuseppe J Delli Carpini G Fichera M Grelloni C. Malignant transformation of postmenopausal endometriosis: A systematic review of the literature. Cancers (2021) 13(16). doi: 10.3390/cancers13164026 PMC8394809 34439184

[4] Rolla E. Endometriosis: advances and controversies in classification, pathogenesis, diagnosis, and treatment. F1000Research (2019) 8. doi: 10.12688/f1000research.14817.1 PMC6480968 31069056

[5] Yamaguchi K Mandai M Toyokuni S Hamanishi J Higuchi T Takakura K. Contents of endometriotic cysts, especially the high concentration of free iron, are a possible cause of carcinogenesis in the cysts through the iron-induced persistent oxidative stress. Clin Cancer Res (2008) 14(1):32–40. doi: 10.1158/1078-0432.CCR-07-1614 18172249

[6] Katagiri H Nakayama K Razia S Nakamura K Sato EMI Ishibashi T. Loss of autophagy-related protein Beclin 1 may define poor prognosis in ovarian clear cell carcinomas. Int J Oncol (2015) 47(6):2037–44. doi: 10.3892/ijo.2015.3191 PMC4665333 26458502

[7] Pearce CL Templeman C Rossing MA Lee A Near AM Webb PM. Association between endometriosis and risk of histological subtypes of ovarian cancer: a pooled analysis of case–control studies. Lancet Oncol (2012) 13(4):385–94. doi: 10.1016/S1470-2045(11)70404-1 PMC3664011 22361336

[8] Dawson A Llauradó Fernandez M Anglesio M Yong PJ Carey MS. Endometriosis and endometriosis-associated cancers: new insights into the molecular mechanisms of ovarian cancer development. ecancermedicalscience (2018) 12. doi: 10.3332/ecancer.2018.803 PMC5813919 29456620

[9] Edwards RP Huang X Vlad AM. Chronic inflammation in endometriosis and EAOC: New roles for the “old” complement pathway. OncoImmunology (2015) 4(5). doi: 10.1080/2162402X.2014.1002732 PMC4485759 26155393

[10] Barreta A Sarian LO Ferracini AC Costa LBE Mazzola PG de Angelo Andrade L. Immunohistochemistry expression of targeted therapies biomarkers in ovarian clear cell and endometrioid carcinomas (type I) and endometriosis. Hum Pathol (2019) 85:72–81. doi: 10.1016/j.humpath.2018.10.028 30447298

[11] Guan B Wang T-L Shih I-M. ARID1A, a factor that promotes formation of SWI/SNF-mediated chromatin remodeling, is a tumor suppressor in gynecologic cancers. Cancer Res (2011) 71(21):6718–27. doi: 10.1158/0008-5472.CAN-11-1562 PMC3206175 21900401

[12] Wiegand KC Shah SP Al-Agha OM Zhao Y Tse K Zeng T. ARID1AMutations in endometriosis-associated ovarian carcinomas. New Engl J Med (2010) 363(16):1532–43. doi: 10.1056/NEJMoa1008433 PMC2976679 20942669

[13] Kolasa IK Rembiszewska A Janiec-Jankowska A Dansonka-Mieszkowska A Lewandowska AM Konopka B. PTEN mutation, expression and LOH at its locus in ovarian carcinomas. Relation to TP53, K-RAS and BRCA1 mutations. Gynecologic Oncol (2006) 103(2):692–7. 10.1016/j.ygyno.2006.05.007 16793127

[14] DeVorkin L Hattersley M Kim P Ries J Spowart J Anglesio MS. Autophagy inhibition enhances sunitinib efficacy in clear cell ovarian carcinoma. Mol Cancer Res (2017) 15(3):250–8. doi: 10.1158/1541-7786.MCR-16-0132 PMC5451253 28184014

[15] Tang FH Hsieh TH Hsu CY Lin HY Long CY Cheng KH. KRAS mutation coupled with p53 loss is sufficient to induce ovarian carcinosarcomas in mice. Int J Cancer (2017) 140(8):1860–9. doi: 10.1002/ijc.30591 28032649

[16] Ye S Yang J You Y Cao D Huang H Wu M. Clinicopathologic significance of HNF-1β, AIRD1A, and PIK3CA expression in ovarian clear cell carcinoma. Medicine (2016) 95(9). doi: 10.1097/MD.0000000000003003 PMC4782907 26945423

[17] Khalique S Nash S Natrajan R. Definitive study shows no association between ARID1A mutation status and clinical outcome in endometriosis-related ovarian cancers‡. J Pathol (2022) 258(1):1–3. 35647895 10.1002/path.5973 PMC9540905

[18] Yachida N Yoshihara K Suda K Nakaoka H Ueda H Sugino K. ARID1A protein expression is retained in ovarian endometriosis with ARID1A loss-of-function mutations: implication for the two-hit hypothesis. Sci Rep (2020) 10(1). doi: 10.1038/s41598-020-71273-7 PMC7459315 32868822

[19] Ayhan A Kuhn E Wu R-C Ogawa H Bahadirli-Talbott A Mao T-L. CCNE1 copy-number gain and overexpression identify ovarian clear cell carcinoma with a poor prognosis. Modern Pathol (2017) 30(2):297–303. doi: 10.1038/modpathol.2016.160 27767100

[20] Bosse T ter Haar NT Seeber LM Diest P Hes FJ Vasen HFA. Loss of ARID1A expression and its relationship with PI3K-Akt pathway alterations, TP53 and microsatellite instability in endometrial cancer. Modern Pathol (2013) 26(11):1525–35. doi: 10.1038/modpathol.2013.96 23702729

[21] Feroze-Zaidi F Fusi L Takano M Higham J Salker MS Goto T. Role and regulation of the serum- and glucocorticoid-regulated kinase 1 in fertile and infertile human endometrium. Endocrinology (2007) 148(10):5020–9. doi: 10.1210/en.2007-0659 17640988

[22] Kuo K-T Mao T-L Jones S Veras E Ayhan A Wang T-L. Frequent activating mutations of PIK3CA in ovarian clear cell carcinoma. Am J Pathol (2009) 174(5):1597–601. doi: 10.2353/ajpath.2009.081000 PMC2671248 19349352

[23] Gounaris I Charnock-Jones DS Brenton JD. Ovarian clear cell carcinoma-bad endometriosis or bad endometrium? J Pathol (2011) 225(2):157–60. doi: 10.1002/path.2970 21898874

[24] Yodoi J Yamashita Y Akatsuka S Shinjo K Yatabe Y Kobayashi H. Met is the most frequently amplified gene in endometriosis-associated ovarian clear cell adenocarcinoma and correlates with worsened prognosis. PloS One (2013) 8(3). doi: 10.1371/journal.pone.0057724 PMC3587638 23469222

[25] Munksgaard PS Blaakaer J. The association between endometriosis and ovarian cancer: A review of histological, genetic and molecular alterations. Gynecologic Oncol (2012) 124(1):164–9. doi: 10.1016/j.ygyno.2011.10.001 22032835

[26] Mandai M Yamaguchi K Matsumura N Baba T Konishi I. Ovarian cancer in endometriosis: molecular biology, pathology, and clinical management. Int J Clin Oncol (2009) 14(5):383–91. doi: 10.1007/s10147-009-0935-y 19856044

[27] Kurman RJ Shih I-M. Molecular pathogenesis and extraovarian origin of epithelial ovarian cancer—Shifting the paradigm. Hum Pathol (2011) 42(7):918–31. doi: 10.1016/j.humpath.2011.03.003 PMC3148026 21683865

[28] Taniguchi F Itamochi H Harada T Terakawa N. Fibroblast growth factor receptor 2 expression may be involved in transformation of ovarian endometrioma to clear cell carcinoma of the ovary. Int J Gynecologic Cancer (2013) 23(5):791–6. doi: 10.1097/IGC.0b013e31828f38c4 23640291

[29] Steele IA Edmondson RJ Leung HY Davies BR. Ligands to FGF receptor 2-IIIb induce proliferation, motility, protection from cell death and cytoskeletal rearrangements in epithelial ovarian cancer cell lines. Growth Factors (2009) 24(1):45–53. 10.1080/08977190500361697 16393693

[30] Braza-Boïls A Marí-Alexandre J Gilabert J Sánchez-Izquierdo D España F Estellés A. MicroRNA expression profile in endometriosis: its relation to angiogenesis and fibrinolytic factors. Hum Reproduction (2014) 29(5):978–88. doi: 10.1093/humrep/deu019 24608518

[31] Matsuzaki S Darcha C. Epithelial to mesenchymal transition-like and mesenchymal to epithelial transition-like processes might be involved in the pathogenesis of pelvic endometriosis†. Hum Reproduction (2012) 27(3):712–21. doi: 10.1093/humrep/der442 22215621

[32] Braza-Boïls A Salloum-Asfar S Marí-Alexandre J Arroyo AB González-Conejero R Barceló-Molina M. Peritoneal fluid modifies the microRNA expression profile in endometrial and endometriotic cells from women with endometriosis. Hum Reproduction (2015) 30(10):2292–302. doi: 10.1093/humrep/dev204 26307093

[33] Wang L Huang W Ren C Zhao M Jiang X Fang X. Analysis of serum microRNA profile by solexa sequencing in women with endometriosis. Reprod Sci (2016) 23(10):1359–70. doi: 10.1177/1933719116641761 27412772

[34] Zhao M Tang Q Wu W Xia Y Chen D Wang X. miR-20a contributes to endometriosis by regulating NTN4 expression. Mol Biol Rep (2014) 41(9):5793–7. doi: 10.1007/s11033-014-3452-7 24972566

[35] Cosar E Mamillapalli R Ersoy GS Cho S Seifer B Taylor HS. Serum microRNAs as diagnostic markers of endometriosis: a comprehensive array-based analysis. Fertility Sterility (2016) 106(2):402–9. doi: 10.1016/j.fertnstert.2016.04.013 27179784

[36] Cho S Mutlu L Grechukhina O Taylor HS. Circulating microRNAs as potential biomarkers for endometriosis. Fertility Sterility (2015) 103(5):1252–60.e1. doi: 10.1016/j.fertnstert.2015.02.013 25772772 PMC4417410

[37] Kashiwagi M Tortorella M Nagase H Brew K. TIMP-3 is a potent inhibitor of aggrecanase 1 (ADAM-TS4) and aggrecanase 2 (ADAM-TS5). J Biol Chem (2001) 276(16):12501–4. doi: 10.1074/jbc.C000848200 11278243

[38] Burns KA Thomas SY Hamilton KJ Young SL Cook DN Korach KS. Early endometriosis in females is directed by immune-mediated estrogen receptor α and IL-6 cross-talk. Endocrinology (2018) 159(1):103–18. doi: 10.1210/en.2017-00562 PMC5761597 28927243

[39] Scutiero G Iannone P Bernardi G Bonaccorsi G Spadaro S Volta CA. Oxidative stress and endometriosis: A systematic review of the literature. Oxid Med Cell Longevity (2017) 2017:1–7. doi: 10.1155/2017/7265238 PMC5625949 29057034

[40] Kobayashi H. Potential scenarios leading to ovarian cancer arising from endometriosis. Redox Rep (2016) 21(3):119–26. doi: 10.1179/1351000215Y.0000000038 PMC6837701 26317761

[41] Marí A Pellín C Moreno M García O Calabuig F Gilabert-Estellés J. Interplay between microRNAs and oxidative stress in ovarian conditions with a focus on ovarian cancer and endometriosis. Int J Mol Sci (2019) 20(21). doi: 10.3390/ijms20215322 PMC6862266 31731537

[42] Sanchez M Torres JV Tormos C Iradi A Muñiz P Espinosa O. Impairment of antioxidant enzymes, lipid peroxidation and 8-oxo-2′-deoxyguanosine in advanced epithelial ovarian carcinoma of a Spanish community. Cancer Letters (2006) 233(1):28–35. doi: 10.1016/j.canlet.2005.02.036 15899547

[43] Limón-Pacheco J Gonsebatt ME. The role of antioxidants and antioxidant-related enzymes in protective responses to environmentally induced oxidative stress. Mutat Research/Genetic Toxicol Environ Mutagenesis (2009) 674(1-2):137–47. doi: 10.1016/j.mrgentox.2008.09.015 18955158

[44] Yoshida S Furukawa N Haruta S Tanase Y Kanayama S Noguchi T. Theoretical model of treatment strategies for clear cell carcinoma of the ovary: Focus on perspectives. Cancer Treat Rev (2009) 35(7):608–15. doi: 10.1016/j.ctrv.2009.07.002 19665848

[45] Kobayashi H. Clear cell carcinoma of the ovary: Potential pathogenic mechanisms (Review). Oncol Rep (2010) 23(5). doi: 10.3892/or_00000750 20372830

[46] Kobayashi H Yamada Y Kanayama S Furukawa N Noguchi T Haruta S. The role of hepatocyte nuclear factor-1β in the pathogenesis of clear cell carcinoma of the ovary. Int J Gynecologic Cancer (2009) 19(3):471–9. doi: 10.1111/IGC.0b013e3181a19eca 19407577

[47] Liu P Khurana A Rattan R He X Kalloger S Dowdy S. Regulation of HSulf-1 expression by variant hepatic nuclear factor 1 in ovarian cancer. Cancer Res (2009) 69(11):4843–50. doi: 10.1158/0008-5472.CAN-08-3065 PMC2713066 19487294

[48] Fiedor J Burda K. Potential role of carotenoids as antioxidants in human health and disease. Nutrients (2014) 6(2):466–88. doi: 10.3390/nu6020466 PMC3942711 24473231

[49] Fujimoto Y Imanaka S Yamada Y Ogawa K Ito F Kawahara N. Comparison of redox parameters in ovarian endometrioma and its Malignant transformation. Oncol Lett (2018). doi: 10.3892/ol.2018.9242 PMC6126218 30214615

[50] Baxter SW. GSTM1 null polymorphism and susceptibility to endometriosis and ovarian cancer. Carcinogenesis (2001) 22(1):63–6. doi: 10.1093/carcin/22.1.63 11159742

[51] Niiro E Kawahara N Yamada Y Yoshimoto C Shimada K Sudo T. Immunohistochemical expression of CD44v9 and 8-OHdG in ovarian endometrioma and the benign endometriotic lesions adjacent to clear cell carcinoma. J Obstetrics Gynaecol Res (2019) 45(11):2260–6. doi: 10.1111/jog.14093 31411797

[52] Wang CT Wang DB Liu KR Li Y Sun CX Guo CS. Inducing Malignant transformation of endometriosis in rats by long-term sustaining hyperestrogenemia and typeII diabetes. Cancer Sci (2014) 106(1):43–50. 25421527 10.1111/cas.12573 PMC4317770

[53] Singh A Gupta S Sachan M. Epigenetic biomarkers in the management of ovarian cancer: current prospectives. Front Cell Dev Biol (2019) 7. doi: 10.3389/fcell.2019.00182 PMC6761254 31608277

[54] Mendizabal I Zeng J Keller TE Yi SV. Body-hypomethylated human genes harbor extensive intragenic transcriptional activity and are prone to cancer-associated dysregulation. Nucleic Acids Res (2017). doi: 10.1093/nar/gkx020 PMC5416765 28115635

[55] Li Y An D Guan Y-x Kang S. Aberrant methylation of the E-cadherin gene promoter region in endometrium and ovarian endometriotic cysts of patients with ovarian endometriosis. Gynecologic Obstetric Invest (2017) 82(1):78–85. doi: 10.1097/IGC.0000000000000021 27023436

[56] Martini M Ciccarone M Garganese G Maggiore C Evangelista A Rahimi S. Possible involvement ofhMLH1, p16INK4a andPTEN in the Malignant transformation of endometriosis. Int J Cancer (2002) 102(4):398–406. doi: 10.2174/13816128113199990540 12402310

[57] Senthong A Kitkumthorn N Rattanatanyong P Khemapech N Triratanachart S Mutirangura A. Differences in LINE-1 methylation between endometriotic ovarian cyst and EAOC. Int J Gynecologic Cancer (2014) 24(1):36–42. 10.1097/IGC.0000000000000021 24304685

[58] Zhou H Li J Podratz K Tipton T Marzolf S Chen H. Hypomethylation and activation of syncytin-1 gene in endometriotic tissue. Curr Pharm Design (2014) 20(11):1786–95. doi: 10.1186/1757-2215-7-73 23888948

[59] Ren F Wang D Jiang Y Ren F. Epigenetic inactivation of hMLH1 in the Malignant transformation of ovarian endometriosis. Arch Gynecol Obstetrics (2011) 285(1):215–21. doi: 10.1242/dev.148296 21556900

[60] Ren F Wang D-B Li T Chen Y-H Li Y. Identification of differentially methylated genes in the Malignant transformation of ovarian endometriosis. J Ovarian Res (2014) 7(1). doi: 10.1016/j.ygyno.2008.09.006 PMC4105232 25298284

[61] Mevel R Draper JE Lie-a-Ling M Kouskoff V Lacaud G. RUNX transcription factors: orchestrators of development. Development (2019) 146(17). doi: 10.1016/j.ygyno.2015.07.009 31488508

[62] Nevadunsky NS Barbieri JS Kwong J Merritt MA Welch WR Berkowitz RS. RUNX3 protein is overexpressed in human epithelial ovarian cancer. Gynecologic Oncol (2009) 112(2):325–30. doi: 10.1089/omi.2009.0030 18937968

[63] Barghout SH Zepeda N Vincent K Azad AK Xu Z Yang C. RUNX3 contributes to carboplatin resistance in epithelial ovarian cancer cells. Gynecologic Oncol (2015) 138(3):647–55. doi: 10.3892/or.2014.3524 26186909

[64] Zhang S Wei L Zhang A Zhang L Yu H. RUNX3 gene methylation in epithelial ovarian cancer tissues and ovarian cancer cell lines. OMICS: A J Integr Biol (2009) 13(4):307–11. doi: 10.1055/s-0029-1242991 19645591

[65] Guo C Ren F Wang D Li YAN Liu K Liu S. RUNX3 is inactivated by promoter hypermethylation in Malignant transformation of ovarian endometriosis. Oncol Rep (2014) 32(6):2580–8. doi: 10.1111/his.12721 25333219

[66] Bulun S Cheng Y-H Pavone M Xue Q Attar E Trukhacheva E. Estrogen receptor-β, estrogen receptor-α, and progesterone resistance in endometriosis. Semin Reprod Med (2010) 28(01):036–43. doi: 10.1016/j.acthis.2014.02.007 PMC3073375 20104427

[67] Nishikimi K Kiyokawa T Tate S Iwamoto M Shozu M. ARID1Aexpression in ovarian clear cell carcinoma with an adenofibromatous component. Histopathology (2015) 67(6):866–71. doi: 10.1186/1476-4598-9-264 25913291

[68] Lin K Zhan H Ma J Xu K Wu R Zhou C. Increased steroid receptor RNA activator protein (SRAP) accompanied by decreased estrogen receptor-beta (ER-β) levels during the Malignant transformation of endometriosis associated ovarian clear cell carcinoma. Acta Histochemica (2014) 116(5):878–82. doi: 10.1007/s00795-004-0252-5 24704270

[69] Schagdarsurengin U Richter AM Hornung J Lange C Steinmann K Dammann RH. Frequent epigenetic inactivation of RASSF2 in thyroid cancer and functional consequences. Mol Cancer (2010) 9(1). doi: 10.1007/s00428-003-0813-3 PMC2956732 20920251

[70] Otsuka J Okuda T Sekizawa A Amemiya S Saito H Okai T. K-ras mutation may promote carcinogenesis of endometriosis leading to ovarian clear cell carcinoma. Med Electron Microscopy (2004) 37(3). doi: 10.1016/j.beem.2015.04.008 15449112

[71] Fauvet R Poncelet C Hugol D Lavaur A Feldmann G Dara E. Expression of apoptosis-related proteins in endometriomas and benign and Malignant ovarian tumours. Virchows Archiv (2003) 443(1):38–43. doi: 10.1080/00016340701619407 12756564

[72] Jia M Dahlman-Wright K Gustafsson J-Å. Estrogen receptor alpha and beta in health and disease. Best Pract Res Clin Endocrinol Metab (2015) 29(4):557–68. 10.1016/j.beem.2015.04.008 26303083

[73] Oxholm D Breth Knudsen U Kryger-Baggesen N Ravn P. Postmenopausal endometriosis. Acta Obstetricia Gynecologica Scandinavica (2007) 86(10):1158–64. doi: 10.1097/GCO.0000000000000548 17851817

[74] Lavery S Gillmer M. Malignant transformation of residual endometriosis in women on unopposed oestrogen hormone replacement therapy. BJOG: Int J Obstetrics Gynaecol (2001) 108(10):1106–7. doi: 10.1016/j.fertnstert.2005.08.017 11702846

[75] Ladanyi C Boyd S Sticco P Mohling S. Postmenopausal endometriosis, where are we now? Curr Opin Obstetrics Gynecol (2019) 31(4):267–78. doi: 10.1016/0002-9378(93)90433-J 31276453

[76] Matsuzaki S Canis M Pouly J-L Déchelotte PJ Mage G. Analysis of aromatase and 17β-hydroxysteroid dehydrogenase type 2 messenger ribonucleic acid expression in deep endometriosis and eutopic endometrium using laser capture microdissection. Fertility Sterility (2006) 85(2):308–13. doi: 10.1111/j.1349-7006.2008.00988.x 16595205

[77] Khorram O Taylor RN Ryan IP Schall TJ Landers DV. Peritoneal fluid concentrations of the cytokine RANTES correlate with the severity of endometriosis. Am J Obstetrics Gynecol (1993) 169(6):1545–9. doi: 10.3390/ijms21093177 7505529

[78] Suzuki F Akahira J-i Miura I Suzuki T Ito K Hayashi S-i. Loss of estrogen receptor β isoform expression and its correlation with aberrant DNA methylation of the 5′-untranslated region in human epithelial ovarian carcinoma. Cancer Sci (2008) 99(12):2365–72. doi: 10.3892/etm.2011.376 PMC11159089 19032364

[79] Kovács T Szabó-Meleg E Ábrahám IM. Estradiol-induced epigenetically mediated mechanisms and regulation of gene expression. Int J Mol Sci (2020) 21(9). doi: 10.1007/s12672-018-0350-9 PMC7246826 32365920

[80] Tanase Y Yamada Y Shigetomi H Kajihara H Oonogi A Yoshizawa Y. Modulation of estrogenic action in clear cell carcinoma of the ovary (Review). Exp Ther Med (2012) 3(1):18–24. doi: 10.1016/j.jri.2016.06.004 22969838 PMC3438704

[81] Andersen CL Boisen MM Sikora MJ Ma T Tseng G Suryawanshi S. The evolution of estrogen receptor signaling in the progression of endometriosis to EAOC. Hormones Cancer (2018) 9(6):399–407. doi: 10.1126/scitranslmed.3010626 30302736 PMC10355926

[82] Grandi G Mueller MD Papadia A Kocbek V Bersinger NA Petraglia F. Inflammation influences steroid hormone receptors targeted by progestins in endometrial stromal cells from women with endometriosis. J Reprod Immunol (2016) 117:30–8. doi: 10.1016/j.rbmo.2021.06.030 27371899

[83] Zhao Y Gong P Chen Y Nwachukwu JC Srinivasan S Ko C. Dual suppression of estrogenic and inflammatory activities for targeting of endometriosis. Sci Trans Med (2015) 7(271). doi: 10.3390/medicina55080477 PMC4790140 25609169

[84] Wang D Guo C Li Y Zhou M Wang H Liu J. Oestrogen up-regulates DNMT1 and leads to the hypermethylation of RUNX3 in the Malignant transformation of ovarian endometriosis. Reprod BioMed Online (2022) 44(1):27–37. doi: 10.1097/AOG.0000000000001387 34799276

[85] Zanello M Borghese G Manzara F Degli Esposti E Moro E Raimondo D. Hormonal replacement therapy in menopausal women with history of endometriosis: A review of literature. Medicina (2019) 55(8). doi: 10.3390/cancers15061708 PMC6723930 31416164

[86] Lee AW Ness RB Roman LD Terry KL Schildkraut JM Chang-Claude J. Association between menopausal estrogen-only therapy and ovarian carcinoma risk. Obstetrics Gynecol (2016) 127(5):828–36. doi: 10.1186/1756-9966-29-4 PMC4892111 27054934

[87] Lee HJ Lee B Choi H Kim T Kim Y Kim YB. Impact of hormone replacement therapy on risk of ovarian cancer in postmenopausal women with *de novo* endometriosis or a history of endometriosis. Cancers (2023) 15(6). doi: 10.3892/mmr.2023.12983 PMC10046182 36980597

[88] MaChado DE Berardo PT Palmero CY Nasciutti LE. Higher expression of vascular endothelial growth factor (VEGF) and its receptor VEGFR-2 (Flk-1) and metalloproteinase-9 (MMP-9) in a rat model of peritoneal endometriosis is similar to cancer diseases. J Exp Clin Cancer Res (2010) 29(1). doi: 10.1016/S0015-0282(02)03189-8 PMC2826344 20085636

[89] Zervou M Papageorgiou L Vlachakis D Spandidos D Eliopoulos E Goulielmos G. Genetic factors involved in the co−occurrence of endometriosis with ankylosing spondylitis (Review). Mol Med Rep (2023) 27(5). doi: 10.3892/mco.2021.2321 PMC10071292 36960867

[90] Kats R Collette T Metz CN Akoum A. Marked elevation of macrophage migration inhibitory factor in the peritoneal fluid of women with endometriosis. Fertility Sterility (2002) 78(1):69–76. doi: 10.1016/j.prp.2018.12.017 12095493

[91] Akashi K Nagashima Y Tabata T Oda H. Immunochemical analysis of iron transporters and M2 macrophages in ovarian endometrioma and clear cell adenocarcinoma. Mol Clin Oncol (2021) 15(2). doi: 10.1186/s12929-015-0128-0 PMC8237161 34194738

[92] Yamada Y Uchiyama T Ito F Kawahara N Ogawa K Obayashi C. Clinical significance of M2 macrophages expressing heme oxygenase-1 in Malignant transformation of ovarian endometrioma. Pathol - Res Practice (2019) 215(4):639–43. doi: 10.2353/ajpath.2006.051365 30567635

[93] Chau L-Y. Heme oxygenase-1: emerging target of cancer therapy. J Biomed Sci (2015) 22(1). doi: 10.1158/1055-9965.EPI-10-1180 PMC4380252 25885228

[94] Was H Cichon T Smolarczyk R Rudnicka D Stopa M Chevalier C. Overexpression of heme oxygenase-1 in murine melanoma. Am J Pathol (2006) 169(6):2181–98. doi: 10.1158/1535-7163.MCT-05-0303 PMC1762485 17148680

[95] Clendenen TV Lundin E Zeleniuch-Jacquotte A Koenig KL Berrino F Lukanova A. Circulating inflammation markers and risk of epithelial ovarian cancer. Cancer Epidemiol Biomarkers Prev (2011) 20(5):799–810. doi: 10.1158/0008-5472.CAN-05-0957 21467242 PMC3089656

[96] Szlosarek PW Grimshaw MJ Kulbe H Wilson JL Wilbanks GD Burke F. Expression and regulation of tumor necrosis factor α in normal and Malignant ovarian epithelium. Mol Cancer Ther (2006) 5(2):382–90. doi: 10.1016/S0163-7827(03)00037-7 16505113

[97] Kulbe H Hagemann T Szlosarek PW Balkwill FR Wilson JL. The inflammatory cytokine tumor necrosis factor-α Regulates chemokine receptor expression on ovarian cancer cells. Cancer Res (2005) 65(22):10355–62. 10.1158/0008-5472.CAN-05-0957 16288025

[98] Murakami M Kudo I. Recent advances in molecular biology and physiology of the prostaglandin E2-biosynthetic pathway. Prog Lipid Res (2004) 43(1):3–35. doi: 10.1007/s00011-010-0220-6 14636669

[99] Chang C-M Wang M-L Lu K-H Yang Y-P Juang C-M Wang P-H. Integrating the dysregulated inflammasome-based molecular functionome in the Malignant transformation of endometriosis-associated ovarian carcinoma. Oncotarget (2017) 9(3):3704–26. doi: 10.1593/neo.121262 PMC5790494 29423077

[100] Rutkowski MJ Sughrue ME Kane AJ Ahn BJ Fang S Parsa AT. The complement cascade as a mediator of tissue growth and regeneration. Inflammation Res (2010) 59(11):897–905. doi: 10.1016/j.autrev.2012.01.005 PMC2945462 20517706

[101] Nunez-Cruz S Gimotty PA Guerra MW Connolly DC Wu Y-Q DeAngelis RA. Genetic and pharmacologic inhibition of complement impairs endothelial cell function and ablates ovarian cancer neovascularization. Neoplasia (2012) 14(11):994–IN1. doi: 10.1038/ni.1923 23226093 PMC3514739

[102] Eisenberg VH Zolti M Soriano D. Is there an association between autoimmunity and endometriosis? Autoimmun Rev (2012) 11(11):806–14. doi: 10.1158/1078-0432.CCR-14-1338 22330229

[103] Ricklin D Hajishengallis G Yang K Lambris JD. Complement: a key system for immune surveillance and homeostasis. Nat Immunol (2010) 11(9):785–97. doi: 10.1158/0008-5472.CAN-17-0240 PMC2924908 20720586

[104] Suryawanshi S Huang X Elishaev E Budiu RA Zhang L Kim S. Complement pathway is frequently altered in endometriosis and EAOC. Clin Cancer Res (2014) 20(23):6163–74. doi: 10.1080/2162402X.2017.1349587 PMC4252715 25294912

[105] Kwak JW Laskowski J Li HY McSharry MV Sippel TR Bullock BL. Complement activation via a C3a receptor pathway alters CD4+ T lymphocytes and mediates lung cancer progression. Cancer Res (2018) 78(1):143–56. doi: 10.1002/cam4.1741 PMC5810934 29118090

[106] Zha H Han X Zhu Y Yang F Li Y Li Q. Blocking C5aR signaling promotes the anti-tumor efficacy of PD-1/PD-L1 blockade. OncoImmunology (2017) 6(10). doi: 10.1016/j.fertnstert.2021.08.032 PMC5665063 29123963

[107] Ghoneum A Afify H Salih Z Kelly M Said N. Role of tumor microenvironment in the pathobiology of ovarian cancer: Insights and therapeutic opportunities. Cancer Med (2018) 7(10):5047–56. doi: 10.3109/13547500903183970 PMC6198242 30133163

[108] Nero C Romito I Spadola S Romito A Turco LC Cosentino F. Infiltrating T lymphocytes and programmed cell death protein-1/programmed death-ligand 1 expression in EAOC. Fertility Sterility (2022) 117(1):160–8. doi: 10.1016/j.bpobgyn.2018.01.010 34656305

[109] Hou Z Sun L Gao L Liao L Mao Y Liu J. Cytokine array analysis of peritoneal fluid between women with endometriosis of different stages and those without endometriosis. Biomarkers (2009) 14(8):604–18. doi: 10.1016/j.molmed.2018.07.004 20001709

[110] Riccio L Santulli P Marcellin L Abrão MS Batteux F Chapron C. Immunology of endometriosis. Best Pract Res Clin Obstetrics Gynaecol (2018) 50:39–49. doi: 10.1016/j.cell.2018.03.073 29506962

[111] Symons LK Miller JE Kay VR Marks RM Liblik K Koti M. The immunopathophysiology of endometriosis. Trends Mol Med (2018) 24(9):748–62. doi: 10.1159/000496178 30054239

[112] Zhang AW McPherson A Milne K Kroeger DR Hamilton PT Miranda A. Interfaces of Malignant and immunologic clonal dynamics in ovarian cancer. Cell (2018) 173(7):1755–69.e22. doi: 10.3802/jgo.2019.30.e63 29754820

[113] Tanase Y Kawaguchi R Uchiyama T Kobayashi H. Long-term follow-up after surgical management for atypical endometriosis: A series of nine cases. Case Rep Oncol (2019) 12(1):76–83. doi: 10.1159/000357819 30792646 PMC6381887

[114] Ñiguez Sevilla I MaChado Linde F Marín Sánchez M Arense JJ Torroba A Nieto Díaz A. Prognostic importance of atypical endometriosis with architectural hyperplasia versus cytologic atypia in EAOC. J Gynecologic Oncol (2019) 30(4). doi: 10.1136/ijgc-2020-001210 PMC6543102 31074246

[115] Taniguchi F Harada T Kobayashi H Hayashi K Momoeda M Terakawa N. Clinical characteristics of patients in Japan with ovarian cancer presumably arising from ovarian endometrioma. Gynecologic Obstetric Invest (2014) 77(2):104–10. doi: 10.1371/journal.pone.0165609 24503885

[116] Shinmura H Yoneyama K Harigane E Tsunoda Y Fukami T Matsushima T. Use of tumor markers to distinguish endometriosis-related ovarian neoplasms from ovarian endometrioma. Int J Gynecologic Cancer (2020) 30(6):831–6. doi: 10.1186/s13048-021-00940-8 PMC7362875 32354795

[117] Batra SK Arakawa N Kobayashi H Yonemoto N Masuishi Y Ino Y. Clinical significance of tissue factor pathway inhibitor 2, a serum biomarker candidate for ovarian clear cell carcinoma. PloS One (2016) 11(10). doi: 10.1002/1878-0261.12884 PMC5087914 27798689

[118] Varga J Reviczká A Háková H Švajdler P Rabajdová M Ostró A. Predictive factors of endometriosis progression into ovarian cancer. J Ovarian Res (2022) 15(1). doi: 10.7314/APJCP.2013.14.9.5409 PMC8751310 35012617

[119] Shin HY Yang W Chay DB Lee Ej Chung JY Kim HS. Tetraspanin 1 promotes endometriosis leading to ovarian clear cell carcinoma. Mol Oncol (2021) 15(4):987–1004. doi: 10.1002/uog.8970 33331115 PMC8024726

[120] Saeng-Anan U Pantasri T Neeyalavira V Tongsong T. Sonographic pattern recognition of endometriomas mimicking ovarian cancer. Asian Pacific J Cancer Prev (2013) 14(9):5409–13. doi: 10.2463/mrms.mp.2016-0149 24175835

[121] Testa AC Timmerman D Van Holsbeke C Zannoni GF Fransis S Moerman P. Ovarian cancer arising in endometrioid cysts: ultrasound findings. Ultrasound Obstetrics Gynecol (2011) 38(1):99–106. doi: 10.1016/j.tjog.2020.01.016 21351179

[122] Tanase Y Kawaguchi R Takahama J Kobayashi H. Factors that differentiate between EAOC and benign ovarian endometriosis with mural nodules. Magnetic Resonance Med Sci (2018) 17(3):231–7. doi: 10.1111/j.1525-1438.2006.00754.x PMC6039776 28824051

[123] Udomsinkul P Triratanachart S Oranratanaphan S. Risk factors for endometriotic-cyst associated ovarian cancer: A case controlled study. Taiwanese J Obstetrics Gynecol (2020) 59(2):269–74. doi: 10.1016/j.ejogrb.2014.11.029 32127149

[124] Kobayashi H Sumimoto K Moniwa N Imai M Takakura K Kuromaki T. Risk of developing ovarian cancer among women with ovarian endometrioma: a cohort study in Shizuoka, Japan. Int J Gynecologic Cancer (2007) 17(1):37–43. doi: 10.1259/bjr.20201441 17291229

[125] Kadan Y Fiascone S McCourt C Raker C Granai CO Steinhoff M. Predictive factors for the presence of Malignant transformation of pelvic endometriosis. Eur J Obstetrics Gynecol Reprod Biol (2015) 185:23–7. doi: 10.3389/fonc.2023.1167088 25522113

[126] Zhang X Li M Tang Z Li X Song T. Differentiation between EAOCs and non- EAOCs based on magnetic resonance imaging. Br J Radiol (2021) 94(1125). doi: 10.3892/etm.2018.5779 PMC9327745 33882252

[127] Perelli F Mattei A Scambia G Cavaliere AF. Editorial: Methods in gynecological oncology. Front Oncol (2023) 13:1167088. doi: 10.3892/mco.2019.1889 36969075 PMC10036035

[128] Kawahara N Yamada Y Ito F Hojo W Iwabuchi T Kobayashi H. Discrimination of Malignant transformation from benign endometriosis using a near−infrared approach. Exp Ther Med (2018). doi: 10.3892/ol.2016.4383 PMC5795507 29456705

[129] Matsubara S Kawahara N Horie A Murakami R Horikawa N Sumida D. Magnetic resonance relaxometry improves the accuracy of conventional MRI in the diagnosis of endometriosis−associated ovarian cancer: A case report. Mol Clin Oncol (2019). doi: 10.3390/biomedicines10112683 PMC6667887 31396388

[130] Iwabuchi T Yoshimoto C Shigetomi H Kobayashi H. Cyst fluid hemoglobin species in endometriosis and its Malignant transformation: The role of metallobiology. Oncol Letters (2016) 11(5):3384–8. doi: 10.3233/CBM-150484 PMC4841012 27123121

[131] Kawahara N Kawaguchi R Maehana T Yamanaka S Yamada Y Kobayashi H. The endometriotic neoplasm algorithm for risk assessment (e-NARA) index sheds light on the discrimination of EAOC from ovarian endometrioma. Biomedicines (2022) 10(11). doi: 10.2463/mrms.mp.2016-0028 PMC9687708 36359203

[132] Yoshimoto C Iwabuchi T Shigetomi H Kobayashi H. Cyst fluid iron-related compounds as useful markers to distinguish Malignant transformation from benign endometriotic cysts. Cancer Biomarkers (2015) 15(4):493–9. doi: 10.3390/cancers13153829 PMC12965089 25835178

[133] Yoshimoto C Takahama J Iwabuchi T Uchikoshi M Shigetomi H Kobayashi H. Transverse relaxation rate of cyst fluid can predict Malignant transformation of ovarian endometriosis. Magnetic Resonance Med Sci (2017) 16(2):137–45. doi: 10.3390/diagnostics12051212 PMC5600073 27646154

[134] Kawahara N Miyake R Yamanaka S. Kobayashi H. A novel predictive tool for discriminating endometriosis associated ovarian cancer from ovarian endometrioma: the R2 predictive index. Cancers (2021) 13(15). doi: 10.1111/aogs.14462 PMC8345171 34359728

[135] Yamanaka S Kawahara N Kawaguchi R Waki K Maehana T Fukui Y. The comparison of three predictive indexes to discriminate Malignant ovarian tumors from benign ovarian endometrioma: the characteristics and efficacy. Diagnostics (2022) 12(5). doi: 10.3802/jgo.2016.27.e31 PMC9140823 35626367

[136] Chao X Wang S Lang J Leng J Fan Q. The application of risk models based on machine learning to predict endometriosis-associated ovarian cancer in patients with endometriosis. Acta Obstetricia Gynecologica Scandinavica (2022) 101(12):1440–9. doi: 10.1016/S1470-2045(20)30061-9 PMC9812095 36210724

[137] Mabuchi S Sugiyama T Kimura T. Clear cell carcinoma of the ovary: molecular insights and future therapeutic perspectives. J Gynecologic Oncol (2016) 27(3). doi: 10.1200/JCO.2011.39.8545 PMC4823362 27029752

[138] Ledermann JA Oza AM Lorusso D Aghajanian C Oaknin A Dean A. Rucaparib for patients with platinum-sensitive, recurrent ovarian carcinoma (ARIEL3): post-progression outcomes and updated safety results from a randomised, placebo-controlled, phase 3 trial. Lancet Oncol (2020) 21(5):710–22. doi: 10.1016/S1470-2045(17)30279-6 PMC8210534 32359490

[139] Alsop K Fereday S Meldrum C deFazio A Emmanuel C George J. BRCA mutation frequency and patterns of treatment response in BRCA mutation–positive women with ovarian cancer: A report from the Australian ovarian cancer study group. J Clin Oncol (2012) 30(21):2654–63. doi: 10.1200/JCO.2015.62.3397 PMC3413277 22711857

[140] Coleman RL Brady MF Herzog TJ Sabbatini P Armstrong DK Walker JL. Bevacizumab and paclitaxel–carboplatin chemotherapy and secondary cytoreduction in recurrent, platinum-sensitive ovarian cancer (NRG Oncology/Gynecologic Oncology Group study GOG-0213): a multicentre, open-label, randomised, phase 3 trial. Lancet Oncol (2017) 18(6):779–91. doi: 10.1093/annonc/mdz135 PMC5715461 28438473

[141] Hamanishi J Mandai M Ikeda T Minami M Kawaguchi A Murayama T. Safety and antitumor activity of anti–PD-1 antibody, nivolumab, in patients with platinum-resistant ovarian cancer. J Clin Oncol (2015) 33(34):4015–22. doi: 10.1200/JCO.18.00283 26351349

[142] Matulonis UA Shapira-Frommer R Santin AD Lisyanskaya AS Pignata S Vergote I. Antitumor activity and safety of pembrolizumab in patients with advanced recurrent ovarian cancer: results from the phase II KEYNOTE-100 study. Ann Oncol (2019) 30(7):1080–7. doi: 10.1016/j.ygyno.2017.06.027 31046082

[143] Latham A Srinivasan P Kemel Y Shia J Bandlamudi C Mandelker D. Microsatellite instability is associated with the presence of lynch syndrome pan-cancer. J Clin Oncol (2019) 37(4):286–95. doi: 10.1002/mc.22325 PMC6553803 30376427

[144] Kawabata A Yanaihara N Nagata C Saito M Noguchi D Takenaka M. Prognostic impact of interleukin-6 expression in stage I ovarian clear cell carcinoma. Gynecologic Oncol (2017) 146(3):609–14. doi: 10.1038/ncomms7118 28673661

[145] Yanaihara N Hirata Y Yamaguchi N Noguchi Y Saito M Nagata C. Antitumor effects of interleukin-6 (IL-6)/interleukin-6 receptor (IL-6R) signaling pathway inhibition in clear cell carcinoma of the ovary. Mol Carcinogenesis (2016) 55(5):832–41. doi: 10.1038/ncb3582 25856562

[146] Chandler RL Damrauer JS Raab JR Schisler JC Wilkerson MD Didion JP. Coexistent ARID1A–PIK3CA mutations promote ovarian clear-cell tumorigenesis through pro-tumorigenic inflammatory cytokine signalling. Nat Commun (2015) 6(1). doi: 10.1158/0008-5472.CAN-19-1302 PMC4308813 25625625

[147] Bitler BG Wu S Park PH Hai Y Aird KM Wang Y. ARID1A-mutated ovarian cancers depend on HDAC6 activity. Nat Cell Biol (2017) 19(8):962–73. 10.1038/ncb3582 PMC5541905 28737768

[148] Fukumoto T Fatkhutdinov N Zundell JA Tcyganov EN Nacarelli T Karakashev S. HDAC6 inhibition synergizes with anti-PD-L1 therapy in ARID1A-inactivated ovarian cancer. Cancer Res (2019) 79(21):5482–9. doi: 10.3892/ol.2015.3268 PMC6825538 31311810

[149] Kobayashi Y Osanai K Tanaka K Nishigaya Y Matsumoto H Momomura M. Endometriotic cyst fluid induces reactive oxygen species (ROS) in human immortalized epithelial cells derived from ovarian endometrioma. Redox Rep (2016) 22(6):361–6. doi: 10.2174/1871520617666170327110712 PMC6837669 27866464

[150] Kobayashi H Shigetomi H Yoshimoto C. Checkpoint kinase 1 inhibitors as targeted molecular agents for clear cell carcinoma of the ovary. Oncol Letters (2015) 10(2):571–6. doi: 10.1016/j.fertnstert.2017.03.013 PMC4508996 26622535

[151] Wang Y Chen Y Fang J. Post-transcriptional and post-translational regulation of central carbon metabolic enzymes in cancer. Anti-Cancer Agents Medicinal Chem (2017) 17(11). doi: 10.1016/j.fertnstert.2016.09.014 28356004

[152] Jørgensen H Hill AS Beste MT Kumar MP Chiswick E Fedorcsak P. Peritoneal fluid cytokines related to endometriosis in patients evaluated for infertility. Fertility Sterility (2017) 107(5):1191–9.e2. doi: 10.1159/000445293 28433374

[153] Vicente-Muñoz S Morcillo I PuChades-Carrasco L Payá V Pellicer A Pineda-Lucena A. Pathophysiologic processes have an impact on the plasma metabolomic signature of endometriosis patients. Fertility Sterility (2016) 106(7):1733–41.e1. doi: 10.1002/ijc.10715 27793377

